# Quality of Life of People with HIV/AIDS in Iran: A Systematic Review and Meta-Analysis

**DOI:** 10.18502/ijph.v49i8.3861

**Published:** 2020-08

**Authors:** Mohammad Reza MALEKI, Naser DERAKHSHANI, Saber AZAMI-AGHDASH, Mehran NADERI, Mahdi NIKOOMANESH

**Affiliations:** 1. Department of Health Services Management, School of Health Management and Information Sciences, Iran University of Medical Sciences, Tehran, Iran; 2. Tabriz Health Services Management Research Center, Health Management and Safety Promotion Research Institute, Tabriz University of Medical Sciences, Tabriz, Iran; 3. Department of Food Science and Technology, Faculty of Nutrition and Food Sciences, Tabriz University of Medical Sciences, Tabriz, Iran; 4. Health Management and Economics Research Center, Iran University of Medical Sciences, Tehran, Iran

**Keywords:** Quality of life, Acquired immunodeficiency syndrome (AIDS), HIV, Iran, Systematic review, Meta-analysis

## Abstract

**Background:**

Assessing the quality of life in HIV/AIDS patients is of great importance not only for evaluating the effect of the disease, but also to measure the impact of the interventions in order to improve their quality of life in clinical researches. Therefore, this study aimed to systematically review the quality of life of HIV/AIDS patients in Iran.

**Methods:**

In this systematic review and meta-analysis, the literature search using the related chain of keywords was conducted from 1 Jan 1987 to 30 Apr 2019 in PubMed, Scopus, Web of Science, Embase, Iranian Scientific Information Database (SID), and Magiran. Moreover, hand search of the key journals and the gray literature was performed. The meta-analysis was performed by CMA2 software.

**Results:**

Out of the 1576 retrieved records, eight studies met the inclusion criteria. The average age of the patients was 37.15 ± 9.46 years. The average score of quality of life before and after sensitivity analysis was (39.13 [28.36–49.901 95% CI *P*>0.000] vs. 49.05 [46.31–51.79 95% CI *P*>0.000]). Moreover, the average score of quality of life was respectively 38.86±3.83 vs. 40±6.37 among married compared with single patients, 56.33±4.67 vs. 43.64±1.94 for employment vs. unemployment status. While quality of life was measured in terms of education level, the score was 29.59±9.34 vs. 41.65±4.45 in the individuals with primary school versus academic education.

**Conclusion:**

The QOL score of the HIV/AIDS patients in Iran was significantly low. Therefore, the study highlights the importance of strengthening efforts to undertake necessary investigations in order to provide adequate health insurance, extensive and affordable welfare services, and more appropriate social and mental supports in order to improve the quality of life of the individuals with HIV/AIDS in Iran.

## Introduction

The human immunodeficiency virus (HIV) infection and the acquired immunodeficiency syndrome (AIDS) are among the most prevalent diseases and should be considered as one of the major challenges for the health systems. These health problems have spread in all countries of the world and to all age groups but 90% of the 37 million infected people are living in developing countries. About 2.5 million children under 15 yr of age are affected by the disease (1–5).

Majority of the infected people with HIV/AIDS are at the age of 25 to 34 yr old. The probability of HIV transmission per exposure to the virus is usually 70% by injection of drug and it can be transmitted to the other 30% by unsafe sexual intercourse, mother-to-child transmission, and blood products or transfusions (6). Similarly, 69.8% of the HIV infected people are those individuals who are struggling with drug abuse and addiction in Iran (7).

The diagnosis of HIV/AIDS severely affects the quality of life (QOL) and the mental health of the individuals (8–11). The health status and the QOL are important indicators of health-related QOL to investigate the patient's compliance with a chronic illness or condition (4, 9, 12). The term “quality of life” or “QOL” can be traced back to the definition of health and a person’s functional status. However, nowadays the WHO defines the QOL as the individual’s perception and judgment of the current life based on the values and culture of the community and by considering the expectations, goals and concerns (13).

The QOL covers several aspects of life in patients with chronic diseases (14) so that the researchers interested in doing a study on QOL, to assess the impact of the disease and to measure the effect of the clinical interventions but few successes have been found for the therapeutic interventions on chronic diseases (1). In addition to the problems that chronic diseases cause to individual health of the patients, they make many challenges in several areas such as families, communities, and governments as a whole (15, 16). Moreover, the progressive nature of HIV/AIDS along with its irrecoverable consequences due to the lack of proper treatment can highly affect the QOL of the patients.

Studying the health-related QOL has been emphasized for assessing the effect of the chronic diseases and the therapeutic interventions but it has indicated that there were few achievements by the interventions (1). Since the prevalence of HIV/AIDS is growing in Iran and regarding the importance of the QOL concept for these patients, it is necessary to identify and analyze the elements that influence their QOL. This way we can be able to prepare the interventions to improve the QOL of these patients and to reduce the disease. The purpose of this study was to systematically review the QOL of HIV/AIDS patients in Iran.

## Methods

This was a systematic review and meta-analysis performed in 2019 according to the book named “A systematic review to support evidence-based medicine” (17–19).

### Search strategy

The required data were gathered by searching from 1 January 1987 to 30 April 2019 in PubMed, Scopus, Web of Science, Embase, Iranian Scientific Information Database (SID), and MagIran along with the Google Scholar search engine. The search key words were “Health-related quality of life”, “quality of life”, “HRQOL”, “QOL”, “HIV”, “Human immunodeficiency virus”, “HIV Infections”, “AIDS”, “Acquired immunodeficiency syndrome” and IRAN. Hand search of the key journals on the issue and the reference check of the included papers were performed. Experts on the field of immunology were also contacted. For the gray literature, the European Association for Grey Literature Exploitation (EAGLE) and Health Care Management Information Consortium (HMIC) was searched. The example literature search was done up in PubMed database (Table 1).

**Table 1:** Complete search strategy for PubMed databases

***Set***	***Strategy***	***Item found***
#1	(((“Health-related quality of life”[Title/Abstract]) OR “quality of life”[Title/Abstract]) OR “HRQOL”[Title/Abstract]) OR “QOL”[Title/Abstract]	232040
#2	((((“HIV”[Title/Abstract]) OR “Human immunodeficiency virus”[Title/Abstract]) OR “HIV Infections”[Title/Abstract]) OR “AIDS”[Title/Abstract]) OR “Acquired immunodeficiency syndrome”[Title/Abstract]	379565
#3	“Iran”[Affiliation]	126417
#4	#1 AND #2 AND #3	26

### Eligibility criteria

#### Inclusion criteria:

- All observational studies (descriptive, cross-sectional, case-control, and cohort) that describe the QOL of the HIV/AIDS patients that were published in Persian and English.

#### Exclusion criteria:

- We excluded abstracts resented at conferences, seminars, newsletters, and letters to editors.

### Review process

Firstly, the titles of the retrieved studies were screened and those that did not match the study purpose were excluded. Then respectively the abstract of the papers and the full-texts were reviewed and assessed in terms of eligibility. The data were extracted by using a researcher-made form piloted by five versions and then revised and finalized. All steps of the data extraction were performed independently by two members of the research team and any disagreement was resolved by discussion or by referring the case to a third person.

### Reporting quality assessment

All eligible studies were assessed by two researchers using the Strengthening the reporting of observational studies in epidemiology checklist (STROBE) (20). The disagreements between the two researchers were referred to as a third person. The checklist was selected because it has been designed specifically for the observational studies and because its Persian translation is validated and available (21). The checklist consists of 22 items (22).

### Data analysis

To calculate the indicators, the meta-analysis was performed using the CMA2 (Comprehensive Meta-Analysis) software. Forest plot was used for reporting the results in which the surface of each square shows the sample size of a particular study and the surrounding lines show the 95% confidence interval (CI). Heterogeneity of the studies was assessed by Q statistics and the I^2^ index. I^2^ above 50% considered as high heterogeneity.

### QOL assessment tools

#### WHO-QOL-BREF

The WHOQOL-BREF instrument is a shorter version of the original instrument developed by the WHO to measure the QOL. It consists of 26 items and four dimensions of physical health, psychological health, social relationships, and environment. The four dimensions contain 24 items and the other two items are related to overall QOL and the overall health-related QOL. The items are scored on a five-point categorical Likert scale from strongly agree=5 to strongly disagree=1. The higher scores on the items indicate the better QOL of the patient. Since the number of the items in each dimension was not equal, the average number of 4 was considered for each dimension. The scores of the dimensions ranged between 4 and 20. The scores then normalized into the range of 0 to 100 (23, 24). Some studies have considered the scores of the items as 0–4. The range of the scores in these studies changed to 0 to 104.

#### SF36

The SF36 tool is a short form Health Survey which consists of 36 items that assess the quality of people’s life by self-reporting. The 36 items are in eight concepts and two main areas of physical health and mental health. The scores by the SF36 range from 0 to 100 in which 0 shows the lowest score and 100 indicates the best quality of life (25).

To get an average score of quality of life of the HIV/AIDS patients in Iran, the reported scores from several studies were adjusted in a way that the scores ranged from 0 to 100.

## Results

**Fig. 1:**
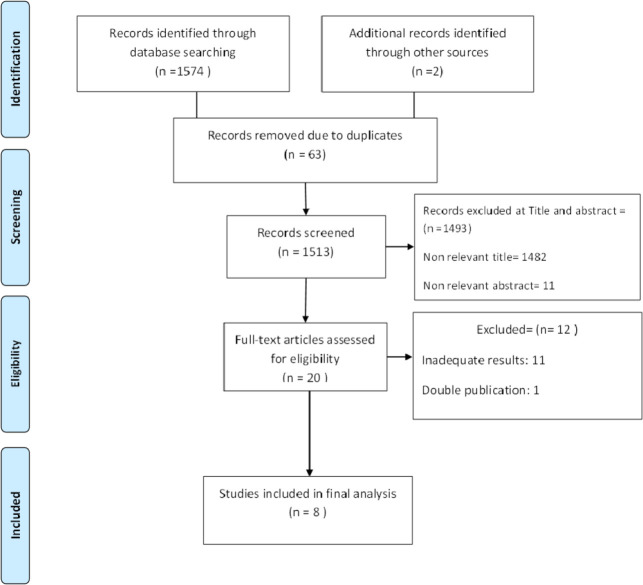
Flow diagram of the search and inclusion process

Out of the 1576 retrieved records from the databases and other sources, 63 were duplicates. Another 1493 records were removed by screening the title and abstract and 12 papers removed as they hadn’t reported the required data. Finally, eight articles met the inclusion criteria and included in the analysis (Fig. 1).

The eight included studies have assessed the QOL of 1547 HIV/AIDS patients in Iran in nine groups. The average age of the participants was 37.15 ± 9.46 years. Characteristics of the included studies are shown in Tables 2 and 3.

**Table 2:** Characteristics of the included studies according to different aspects of QOL

***Reference***	***Tools***	M^*^±SD^**^ Age	***M±SD General***	***M±SD Physical***	***M±SD Psychologic***	***M±SD Social***	***M±SD Environmental***	***M±SD QOL***
(26)	WHO-QOL-Brief	35.4±6.4	3.5±1.34	13.1±4.42	9.2±1.81	6.4±1.6	12.6±2.41	47±6.26
(27)	WHO-QOL-Brief	37.29 ± 8.34	-	47.3±15.7	45.37±17.2	41.44±20.67	45.24±18.68	44.83±18.06
(28)	WHO-QOL-Brief	38.06±9.32	-	11.57±1.83	11.73±3.35	12.08±3.43	12.05±2.67	11.95±2.07
(2)	SF-36	34.9 ± 10.7	48.3±50	32.6±25	46.3±43.7	45.6±43.7	-	47.45±40.6
(29)	SF-36	48.8±19.96	47.5±19.9	50.4±18.9	46.3±17.8	50.4±24.4	55.11±55	48.8±17
(30)	SF-36	38.31±9.15	-	59.9±18.32	46.36±17.31	-	-	53.12±17.81
(31)	WHOQOL	36 ± 6.74	-	-	-	-	-	76.64±16.37
(32)- 1	WHO-QOL-Brief	33.38±7.07	-	11.57±2.56	12.95±2.46	11.13±2.55	21.08±4.5	91.41±15.98
(32)-2	WHO-QOL-Brief	32.28±7.42	-	11.77±2.59	12.40±2.85	11.49±2.63	21.29±4.52	94.98±16.14

**Table 3:** Characteristics of the included studies with consideration to marital status, occupation and literacy in QOL

***Reference***	M^*^±SD^**^ Age	***Marital Status***			***Occupation***			***Literacy***			
		***Mean±SD or (%) Married***	***Mean±SD Or (%) Single***	***Mean±SD Or (%) Widowed***	***Mean±SD Or (%) Separated***	***Mean±SD Or (%) Employed***	***Mean±SD Or (%) Unemployed***	Mean±SD Or (%) Primary^*^	Mean±SD Or (%) Secondary^**^	***Mean±SD Or (%) High school***	***Mean±SD Or (%) Academic***
(26)	35.4±6.4	53.63±7.13	47.20±7.30	40.5±8.82	40.09±7.24	53.82±4.54	43.57±8.31	40.50±7.68	45.78±7.76	52.92±8.77	54.38±4.53
(31)	36±6.74	77.3±16.58	77.32±19.01	69.4±13.67	60.55±4.27	-	-	65.16±18.4	69.77±13.07	76.93±17.1	104.5±8.79
(29)	48.8±19.9	58.1±22.2	46 ± 13.7	46.7±17.5	46.7±17.5	61.1±15.4	45.8±16.1	-	48±16.3	46.6±14.8	24.8±12.4

**Fig. 2:**
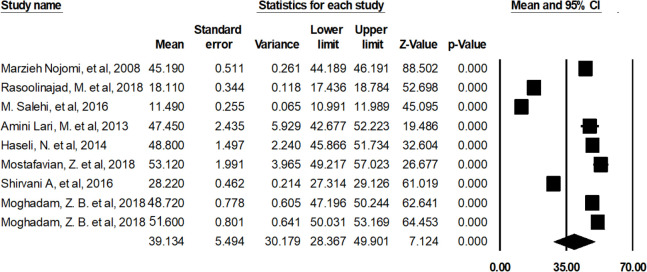
Average score of QOL of HIV/AIDS patients in Iran

The average score of the QOL of HIV/AIDS patients in Iran was 39.13 [95% CI: 28.36-49.901, Q=7255.564, df=8 I2=99.890, *P*>0.000] (Fig. 2). The General dimension had the highest score (32.99) and the Environmental health had the lowest score (18.7) (Table 4).

**Table 4:** QOL of HIV/AIDS patients in Iran according to the QOL dimensions

***Dimensions***	***Dimension statues (95% CI)***				***Heterogeneity test(95% CI)***			
	***Mean***	***Variance***	***Lower limit***	***Upper limit***	***Df***	***Q***	***P-value***	***I^2^***
General	32.995	327.844	−2.493	68.483	2	854.394	0.000	99.766
Physical	30.791	12.047	23.989	37.594	5	1699.754	0.000	99.706
Psychologic	29.124	11.806	22.389	35.858	5	1760.835	0.000	99.716
Social	24.827	10.723	18.409	31.245	4	1248.378	0.000	99.680
Environmental	18.703	4.277	14.650	22.757	3	345.884	0.000	99.133
QOL before sensitive Analysis	39.134	30.179	28.367	49.901	8	7255.564	0.000	99.890

**Fig. 3:**
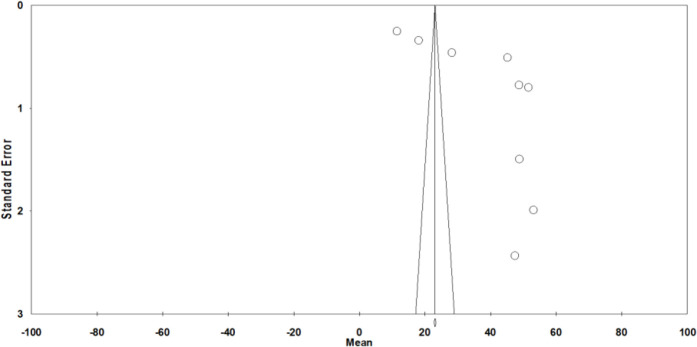
Publication bias of the studies on QOL of the HIV/AIDS patients in Iran

**Fig. 4:**
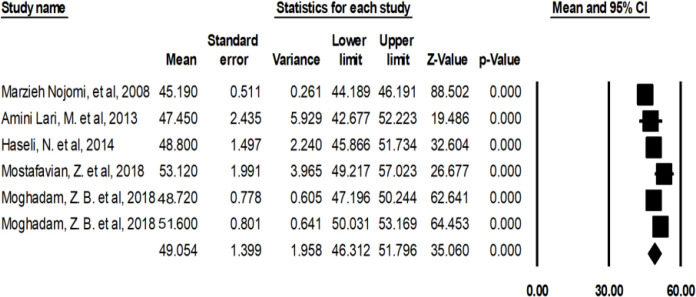
The average score of QOL of HIV/AIDS patients in Iran after the sensitivity analysis

The heterogeneity of the studies was examined by the sensitivity analysis. Three studies (27, 28, 31) were removed from the analysis due to reporting discrete and different results (Fig. 3). The average score of the QOL of the HIV/AIDS patients in Iran after the sensitivity analysis was 49.05 [46.31–51.79 95% CI, Q=57.574, df=5 I^2^=91.316, *P*>0.000] (Fig .4).

**Fig. 5:**
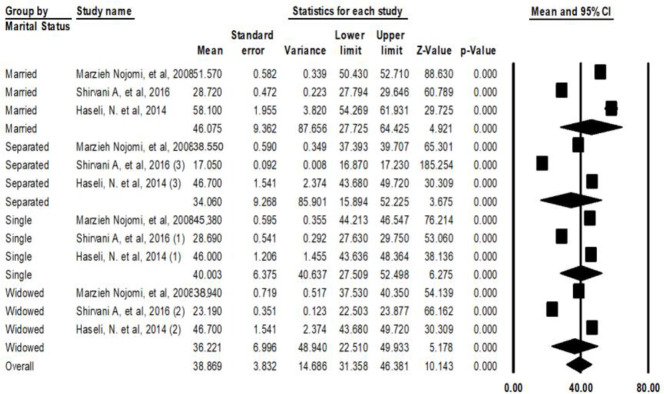
Average scores of QOL of the patients with HIV/AIDS in Iran based on marital status

The married patients had higher QOL with average score of 46.07 [27.725–64.425 95% CI, Q=1.037, df=3 *P*>0.000]. The divorced patients had the lowest QOL scores: 34.06 ± 9.26 (Fig. 5).

**Fig. 6:**
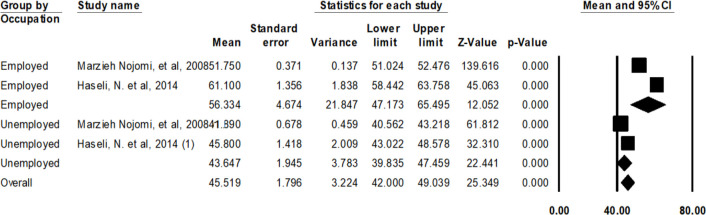
Average scores of QOL of patients with HIV/AIDS in Iran based on employment status

**Fig. 7:**
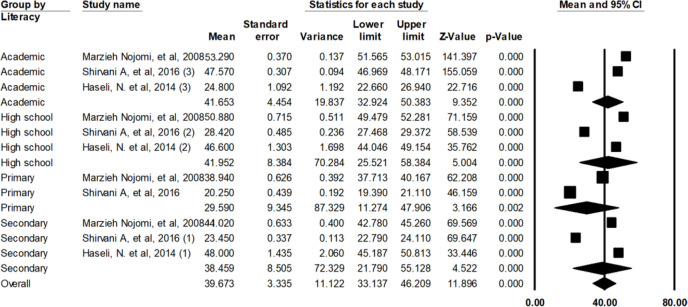
Average scores of QOL of patients with HIV/AIDS in Iran based on education level

The unemployed patients had lower QOL scores with average score of 43.64± 1.94 compare to the employed ones (Fig. 6). As it is seen in Fig. 7, those patients who had lower education (illiterate or primary school) had the lowest average QOL scores (29.59 ± 9.34). The patients with high school education had the highest average QOL scores (41.95 ± 8.3).

## Discussion

This systematic review and meta-analysis included eight studies. The average score of QOL of 1547 HIV/AIDS patients in Iran was calculated to be 39.13. After the sensitivity analysis and removing three studies (27, 28, 31), the numbers changed to 866 patients and the average QOL score of 49.05. The QOL scores were calculated based on educational level, marital status and employment status. The average scores of QOL of the HIV/AIDS patients were 32.99 in General. The average scores have been reported to be respectively 18.7, 30.79, 29.12, and 24.82 in the Environmental, Physical, Psychological and Social dimensions.

The overall QOL scores of the HIV/AIDS patients in Iran were low and unacceptable. The findings about the mentioned dimensions were confirmed in Brazil (33). In Georgia, another study has also reported the QOL score of the HIV/AIDS patients at a very low level, just similar to the findings of this study (34). It is noteworthy to mention the challenges of the daily life including active participation in social life and performing physical activity as the underlying factors of the low QOL scores. These challenges make hardships for the families of the patients and their social life (3, 35–37). Moreover, one of the main reasons for low QOL scores of these patients is the social stigma as many people believe that these people are infected by illegitimate and risky sexual behaviors. Thus, the disease probably carry stigma, patients try to hide their disease and this issue causes many problems. One of the other reasons for the low QOL of some of the HIV/AIDS patients is injecting drugs. In addition to health problems, these patients may face social, economic and mental problems. Due to the growing problems such as poverty, addiction, depression, mental problems, and homelessness many of addicted patients usually do not care so much about their health and do not consider it a priority (37). The findings are in line with a study conducted in Estonia that 60% of participants were injection drug users (38). To prevent and reduce the patients’ social and mental problems, these solutions may be helpful: driving a culture change with the help of media, developing educational programs for these patients, making specific exercise facilities for patients, and providing free consultation services by physicians and psychologists (39–42).

The estimated QOL scores of the HIV/AIDS patients for the married, single, widow, and divorced patients were respectively reported 46.07, 40, 36.22, and 34.06. Therefore, the QOL of the patients was low, especially in divorced ones. A potential reason for this matter might be the mental pressures derived from divorce and the social exclusion by families and the community which severely affects the individuals’ QOL. Similarly, lower scores on social dimension of QOL have been reported for those patients who live alone because of their disease compared to other patients. In contrary, the better QOL scores have been reported for those patients who live with their families and have mental and spiritual support (43, 44). Since the spiritual support by the society and the family is important for the QOL of these patients, providing public education by mass media and specific trainings for the families of the patients may improve the QOL scores of these patients.

Based on educational level, the QOL scores of the HIV/AIDS patients in Iran were estimated to be 41.65 for academic education, 41.95 for high school diploma, 38.45 for middle school (called Guidance School in Iran), and 29.59 for illiterate or primary school. The higher the education of the patients, the higher the QOL score will be. This might be due to indirect effects of education on the QOL. Higher education can also lead to better understanding of the disease and thus the higher QOL (34, 45, 46). Yet, two studies in Brazil reported no significant correlation between the education level and the dimensions of the QOL (44, 47).

The average score of QOL of the HIV/AIDS patients in Iran was 56.33 for employed and 43.64 for unemployed ones. The QOL is significantly affected by the employment status so that the QOL of the unemployed patients was disappointingly low. Other studies also reported similar results in this regard that the unemployed patients and those with lower income have lower QOL score (5, 38, 44, 48–52). HIV/AIDS patients have worse physical function compared to patients with other chronic diseases (53, 54). Employment and having sufficient income influence the most of the dimensions of the QOL of these patients (44, 55–60) so that some researchers have reported the employment as one of the most important determinants of the QOL among these group of patients (37, 43, 44, 61–64). Moreover, more problems and lower physical and mental health status are predictable among unemployed patients (54, 63). However, finding a job is hard for these patients and they may be fired due to the fear of the colleagues from the communicability of the disease (46, 64). Considering the impact of the employment on the QOL of the HIV/AIDS patients, government policies and programs by NGOs should be focused on creating job opportunities for these patients in accordance with their physical and mental conditions, also work-at-home careers and tele-working may improve the QOL of the patients. Moreover, since most of the needs of these patients are common with the other people except for the therapeutic needs, public education programs should be established by the stakeholder organizations to publicize the social requirements of these patients especially their need for employment.

### Limitations

A few numbers of valid studies on the subject due to the nature of the disease and the low participation of the patients should be considered as one of the limitations. Moreover, the variety of the tools used for assessing the QOL of the HIV/AIDS patients in various studies forced the researchers to localize the tools, so that they measured different dimensions of the QOL with no possibility to compare the results with other studies.

## Conclusion

The QOL of HIV/AIDS patients in Iran is improperly low. Therefore, by considering the need for preventing the disease and reducing its consequences, it is necessary to drive a culture change by educating the public about the disease. The governments and/or the related organizations should also contribute to raising the QOL and elimination of the disease by providing specific healthcare packages for these patients. Additionally, due to the hardships that these patients face in the physical, mental and socio-economic aspects of their life, the necessary investigations should be implemented for providing adequate health insurance, more and cheaper welfare services, and more appropriate social and mental supports to improve the quality of life of the HIV/AIDS patients.

## Ethical considerations

Ethical issues (Including plagiarism, informed consent, misconduct, data fabrication and/or falsification, double publication and/or submission, redundancy, etc.) have been completely observed by the authors.
